# pH-Dependent Conformational Changes in Grb2 Monomer Reveal Different Binding Sites for Coumarin: An Insight for Small Molecule Drug Discovery

**DOI:** 10.1021/acsomega.5c09449

**Published:** 2026-01-30

**Authors:** Giovana Casteluci, Raphael Vinicius Rodrigues Dias, Jéssica Andrade Tedesco, Lucas Eduardo Gouveia, Aline Sebastiane Gonçales Ramos de Oliveira, Ícaro Putinhon Caruso, Fernando Alves de Melo

**Affiliations:** † Department of Physics, 135132 São Paulo State University (UNESP), Institute of Biosciences, Humanities and Exact Sciences, 15054-000 São José do Rio Preto, SP Brazil; ‡ Multiuser Center for Biomolecular Innovation (CMIB), São Paulo State University (UNESP), Institute of Biosciences, Humanities and Exact Sciences, 15054-000 São José do Rio Preto, SP Brazil

## Abstract

Growth factor receptor-bound protein 2 (Grb2) is an essential adaptor protein that mediates activation of the RAS/mitogen-activated protein kinase (MAPK) signaling cascade by linking receptor tyrosine kinase to the guanine nucleotide exchange factor SOS (Son of Sevenless). Grb2 exists in a dynamic monomer–dimer equilibrium, with only the monomer competent for signaling. Dysregulation of this pathway is common in cancer, making Grb2 a compelling therapeutic target. Coumarin, a plant-derived compound with reported anticarcinogenic activity, was previously shown to bind the SH2 domain of dimeric Grb2. Here, we investigated the interaction between coumarin and the monomeric form of Grb2 under two physiologically relevant pH conditions (7 and 8), corresponding to healthy and cancer cell environments, respectively. Fluorescence quenching, dynamic light scattering, and STD-NMR analyses revealed that pH modulates Grb2’s conformation, increasing its hydrodynamic diameter and exposing alternative ligand-binding sites. The interaction mapping identified Trp60 (buried in the dimeric form) as a key contact residue and at pH 8.0, coumarin binds Grb2 with a 10-fold higher stability. These findings demonstrate, for the first time, that coumarin interacts with monomeric Grb2 and that pH-sensitive structural dynamics modulate ligand binding. Our results highlight the importance of mimicking disease-relevant physicochemical conditions in ligand discovery efforts and support the development of pH-sensitive inhibitors targeting aberrant MAPK signaling in cancer.

## Graphical abstract

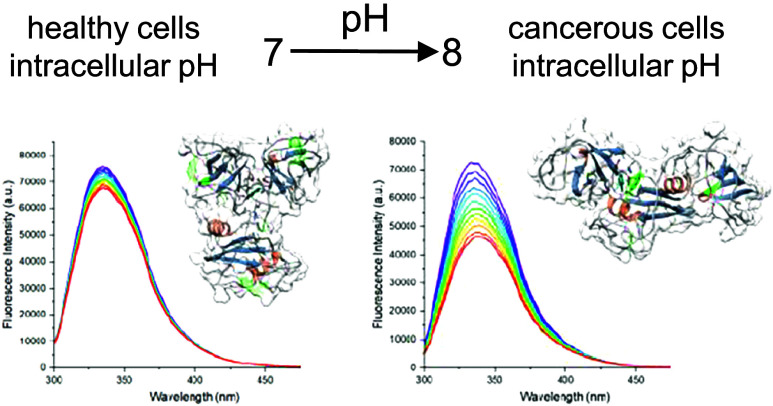

## 1 Introduction

The Growth factor receptor-bound protein 2 (Grb2) allows signaling downstream of the MAPK (Mitogen Activated Protein Kinases) pathway through interactions with active growth factor receptors and the guanine exchange factor protein Son of Sevenless (SOS).^1–3^ Despite being an adaptor protein, Grb2 regulates the activation of the signaling pathway through a monomer–dimer equilibrium, in which the monomer recruits SOS to the membrane and the dimer is its autoinhibited form.^4^

Since Grb2 acts as a bridge between receptor tyrosine kinase and RAS to activate the MAPK cascade, it presents significant potential for inhibiting the signaling pathway in cases of aberrant signaling, such as cancer—a multifactorial disease caused by an uncontrolled growth and proliferation of the cells.^5^

Coumarin (1,2-benzopyrone) is a product of the secondary metabolism of plants that has well-documented anticarcinogenic properties in the literature, such as the inhibition of tumor formation in mice and the inhibition of proliferative potential and induction of apoptosis in human colon and lung cancer cells.^6–8^ Moreover coumarin meets several requirements valued for drug research and development, such as simple structure, high bioavailability, low molecular weight, high solubility in organic solvents, and low toxicity (tolerable daily intake ∼0.1 mg/kg/day).^9–11^

Our research group previously characterized that coumarin interacts with Grb2 through the SH2 domain, more specifically within a hydrophobic pocket near tryptophan 121.^12^ A key limitation of this earlier work, however, is that the study was conducted exclusively on the protein’s autoinhibited, dimeric form. We also recently identified that changing the pH from 7 to 8 induces conformational changes in Grb2 structure.^13^ While the cytosolic pH typically ranges from 7.2–7.4 in healthy cells and 7.6–7.8 in cancerous cells,^14^ we utilized pH 7 and 8 as representative boundary conditions to model these distinct physiological states. In this context, we aimed to investigate the interaction of coumarin with the Grb2 monomer under these specific pH values to determine how cellular alkalization interferes with protein–ligand binding. Our findings indicate that the interaction between Grb2 and coumarin is 10-fold more favorable at pH 8, which is close to the pH value of the intracellular environment in cancer cells. Furthermore, we identified that increasing the pH interferes with the monomer conformation, leading coumarin to bind at different sites. These results confirm that pH can modulate Grb2 interaction with ligands and highlight the importance of choosing appropriate experimental conditions during the molecule testing processes.

## 2 Results and Discussion

As aforementioned, only the Grb2 monomer promotes signaling downstream by interacting with SOS. Thus, we used the Grb2 mutant Y160F in this work due to its ability to prevail as a monomer in solution while retaining the flexibility and net charge of the wild-type protein.^15^ To investigate the protein–ligand interaction, we performed steady-state fluorescence spectroscopy experiments and titrated coumarin in a solution with Grb2 until it reached a concentration ratio of 1:1 (see Section 4.2 of material and methods for details). The interaction was monitored through intrinsic tryptophan fluorescence, as Grb2 has 5 tryptophans: 1 in the N-SH3 domain (Trp36), 2 in the SH2 domain (Trp60 and Trp121), and 2 in the C-SH3 domain (Trp193 and Trp194).^16^

Fluorescence quenching confirmed the interaction between the monomeric Grb2 and coumarin. While the quenching behavior was consistent across the tested temperatures within a given pH condition, a significant difference in the quenching pattern was observed between pH 7 and pH 8 (Figure 1). As fluorescence quenching occurs when the local environment of the tryptophan residue changes, and it is related to the distance between the ligand and the probe,^17^ these results suggest that coumarin might bind into different sites on the protein depending on the pH. This hypothesis is further supported by the association constant (*K*_a_) at pH 8, which is an order of magnitude higher compared to pH 7 (Table 1), as well as our previous findings of the impact of pH on the conformational changes and flexibility of Grb2.^13^

**Figure 1:**
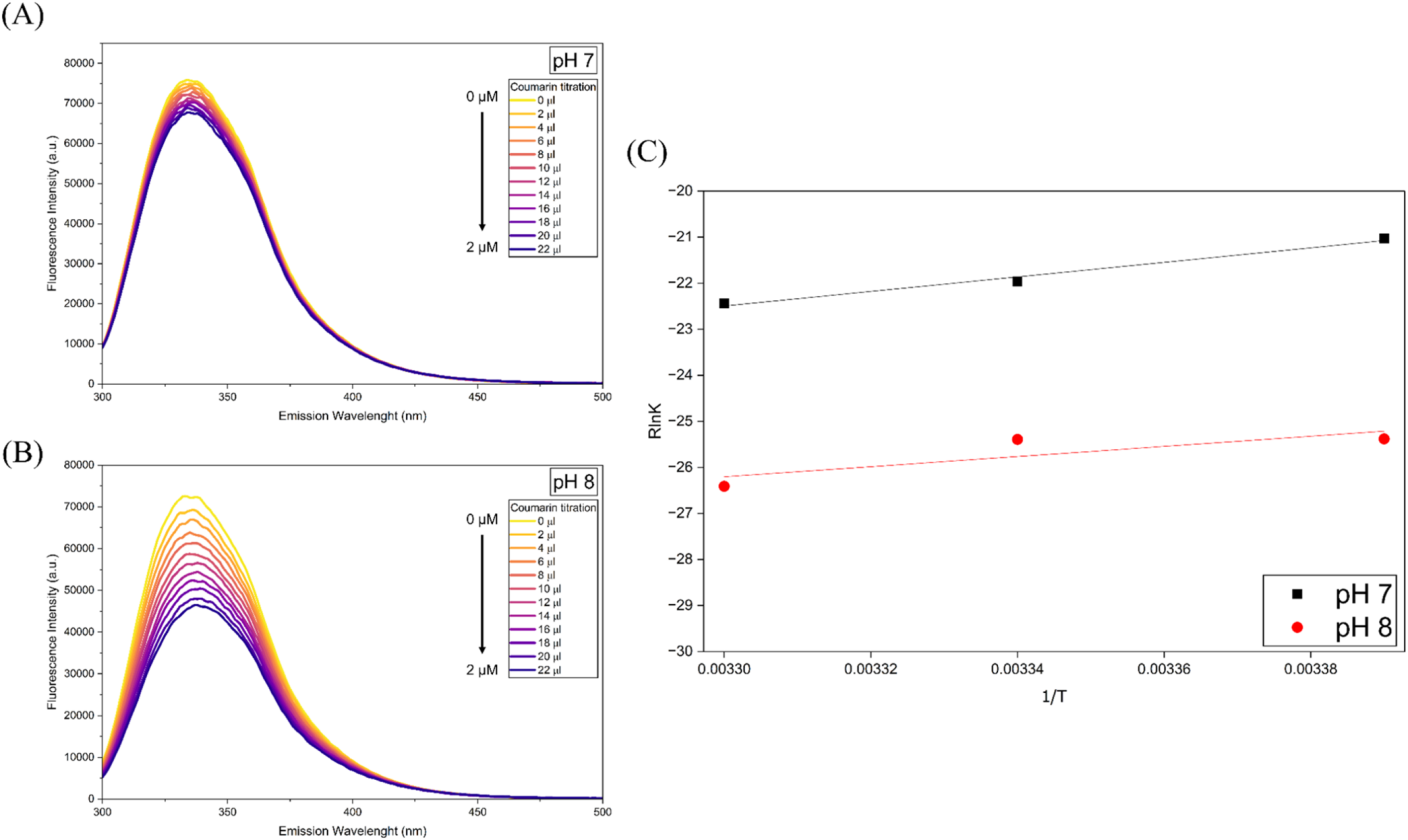
Analysis of fluorescence emission spectra from the monomeric Grb2 and coumarin interaction. Coumarin was titrated until the system reached a 1:1 ratio between protein and ligand. Experiments were performed at 295, 299, and 303 K. Since the data showed a similar quenching pattern across all temperatures, only the results at 299 K are shown here; data for 295 and 303 K are shown in the Supporting Information (Figure S1). (A, B) Fluorescence emission spectra at pH 7 and 8, respectively. (C) Van’t Hoff plot of both pH conditions. The plot was constructed by plotting the natural logarithm of the association constant against the reciprocal of the absolute temperature (1/*T*). The thermodynamic parameters were obtained from the linear regression.

**Table 1:** Association Constant (*K*_a_), Number of Binding Sites (*n*), and Thermodynamic Parameters Obtained from van’t Hoff Analysis for the Grb2-Coumarin Interaction at 295, 299, and 303 K^a^

pH	⟨*K*_a_⟩ (M^–1^)	⟨*n*⟩	⟨Δ*G*⟩ (kcal/mol)	Δ*H* (kcal/mol)	⟨*T*Δ*S*⟩ (kcal/mol)
7	6.4 (±2) × 10^4^	0.85 (±0.1)	–6.5 (±0.3)	15.8	–22.3 (±0.3)
8	4.5 (±1.4) × 10^5^	1.0 (±0.1)	–7.7 (±0.3)	11.5	–19.1 (±0.3)

a The results suggest a favorable (Δ*G* < 0) and entropically driven interaction guided by hydrophobic forces, consistent with the hydrophobic nature of the coumarin molecule.

Further, we performed STD-NMR experiments to map out the coumarin epitopes. In this experiment, when a ligand binds to a protein, the hydrogen protons that are in close proximity (typically within 5–10 Å) to the protein will experience a transfer of magnetization via the Nuclear Overhauser Effect mechanism, which generates the STD signal observed in the difference spectrum (Figure 2).^18^ Thus, the protons that are close to the protein’s binding site will experience a stronger magnetization transfer and show a higher intensity in the STD spectrum. Therefore, if our hypothesis was correct, the epitopes found for each pH condition would present significant differences in relation to magnetization transfer and impact in the STD amplification factor, which indicates the spatial orientation of coumarin in the binding site.

**Figure 2:**
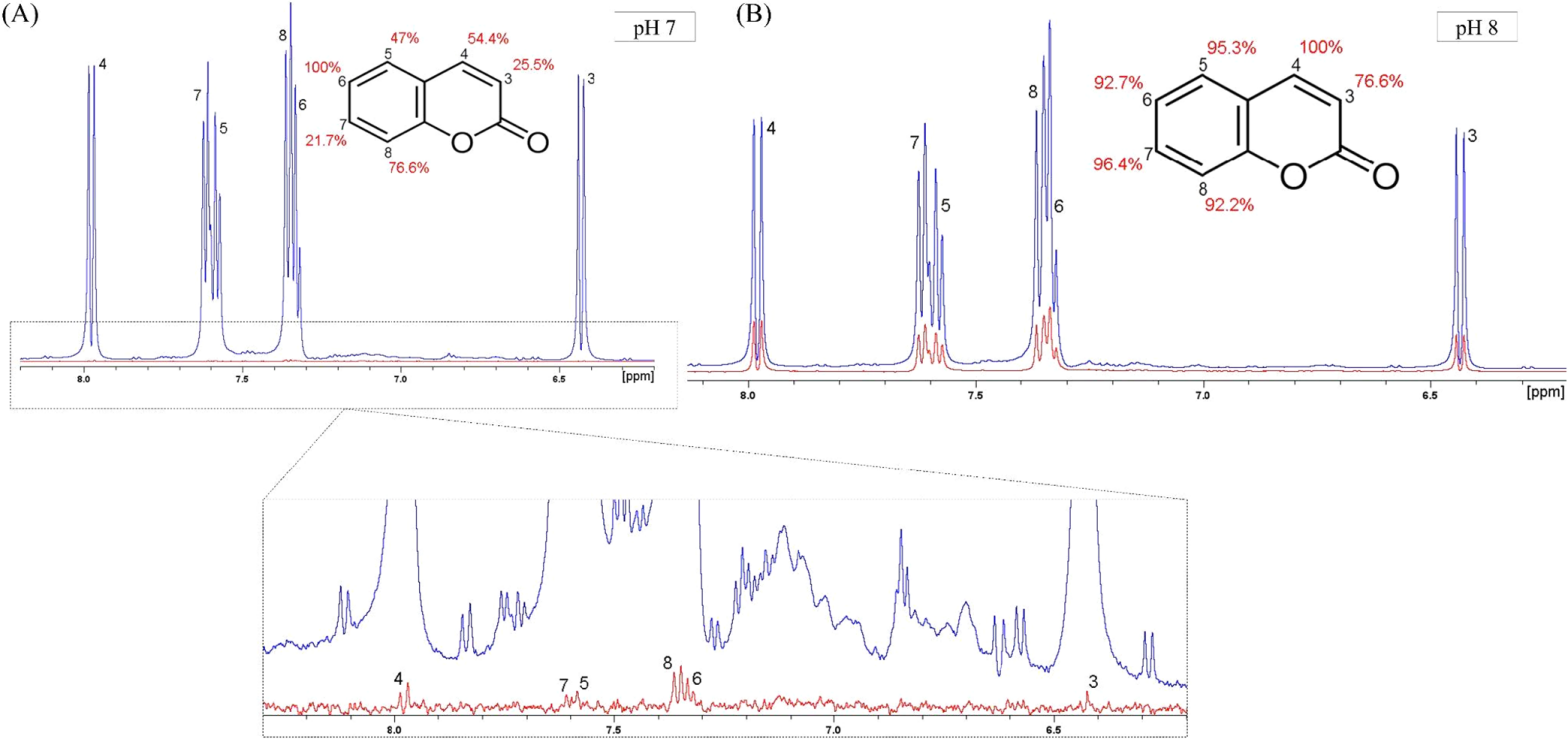
^1^H NMR spectra of Grb2-coumarin off-resonance (blue) and *I*_STD_ (red) spectra. (A) Experiment performed at pH 7. (B) Experiment performed at pH 8.

According to the epitope mapping, at pH 7 only the hydrogens 6 and 8 are closer to the binding site in the protein whereas at pH 8 all the hydrogens present amplification factor values >90%, with hydrogens 4 and 7 being the closest to the protein (Table 2). These results support our hypothesis, suggesting that at pH 7, coumarin might bind to a more flexible or broader region of Grb2, as indicated by the consistently low epitope percentages across the molecule. In contrast, at pH 8, coumarin might be buried within a hydrophobic pocket, as reflected by the higher epitope percentages observed.

**Table 2:** Hydrogen Position, 1D ^1^H-NMR Coumarin Chemical Shift, Intensity of the Difference Spectrum (*I*_STD_) and STD Amplification Factor (*A*_STD_)­^a^

		pH 7		pH 8	
^1^H	δ (ppm)	*I*_STD_	*A*_STD_ (%)	*I*_STD_	*A*_STD_ (%)
3	6.43	0.00155	25.5	0.147	76.6
4	7.98	0.00330	54.4	0.192	100
5	7.56	0.00285	47	0.183	95.3
6	7.31	0.00607	100	0.178	92.7
7	7.62	0.00132	21.7	0.185	96.4
8	7.37	0.00465	76.6	0.177	92.2

a The difference of *I*_STD_ from both pHs corroborates with our hypothesis that Coumarin binds in two different pockets in monomeric Grb2.

Based on the experimental data, we explored potential interaction sites through molecular docking using the monomeric Grb2 structural conformation we obtained in our previous work^15^ and identified two main sites. Site I (Figure 3A) is positioned at the interface between the SH3 and SH2 domains, residing within a highly flexible region composed primarily of random coils. Coumarin binds centrally in this area, and although the docking energy is favorable (−25 kJ) and in close proximity to the residue Trp60, no direct interaction occurs between the ligand and this residue. In contrast, Site II (Figure 3B) is located entirely within the SH2 domain and is structurally more rigid. Coumarin binds within a deep hydrophobic pocket formed between an α helix and a β-sheet, yielding a superior docking energy (−30 kJ), and establishes direct contact with residue Trp60.

**Figure 3:**
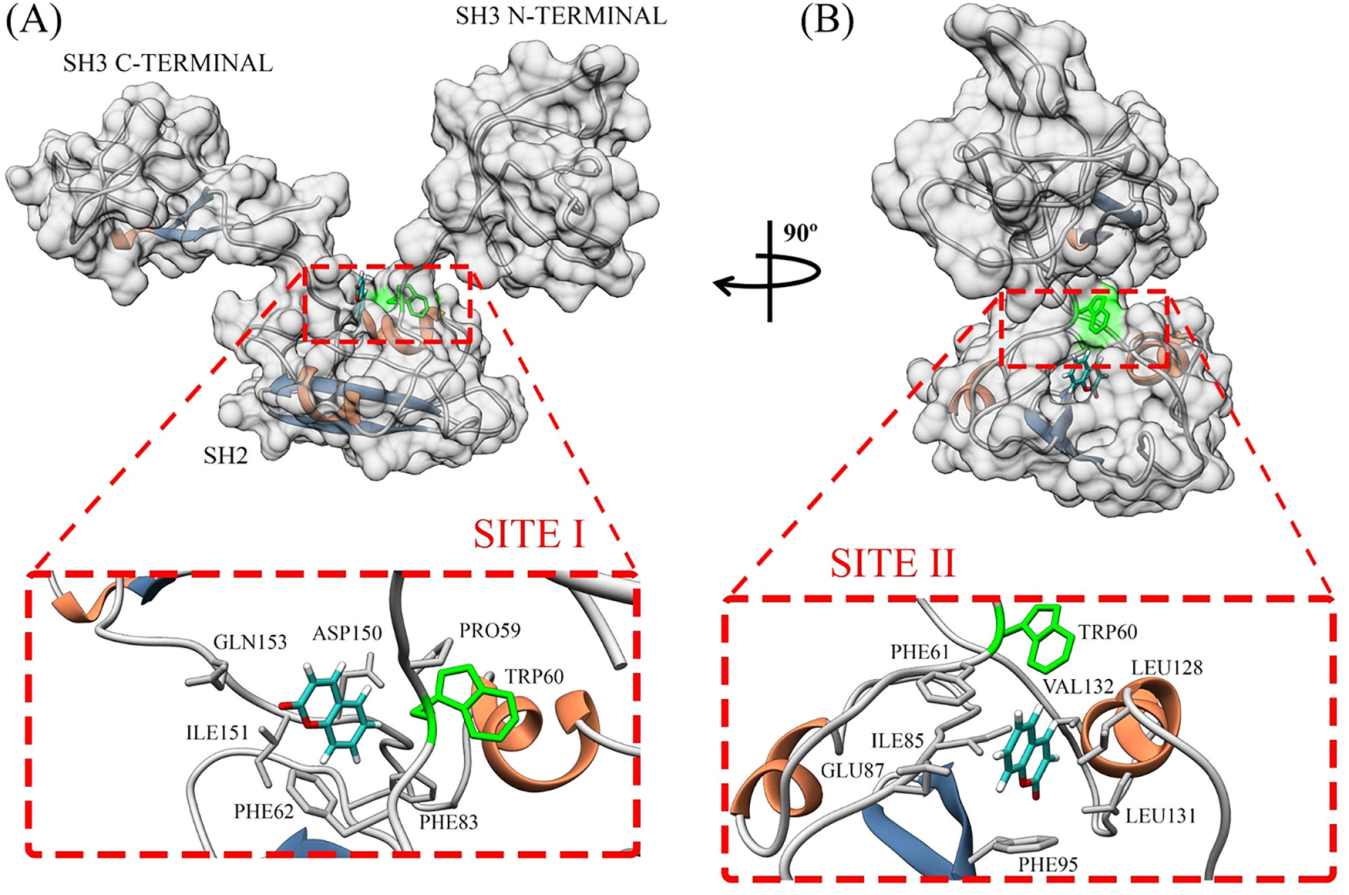
Cartoon representation of the most probable interaction sites for coumarin binding to Grb2 as predicted by molecular docking. The molecular surface of the Grb2 monomer is shown in gray to enhance visualization of the binding sites, with Trp60 highlighted in green. Coumarin is shown in cyan. The inset highlights residues located within 3.4 Å of the ligand. Representations (A) and (B) depict frontal and lateral views of the protein, respectively.

To test the hypothesis that Trp60 is the primary interaction site for coumarin, we performed fluorescence spectroscopy experiments with the additional mutation W60A (Figure S2). The tryptophan-to-alanine substitution was specifically designed to preserve the hydrophobic nature of residue 60. Although the data indicated that coumarin still binds to the mutant (likely involving Trp121, consistent with our previous work on the Grb2 dimer^12^), the experiments revealed a substantial reduction in the association constant under both pH conditions. Specifically, at pH 7, the affinity decreased from 6.4 (±2) × 10^4^ M^–1^ to 3.0 (±0) × 10^4^ M^–1^, representing a 53.6% reduction. A more dramatic effect was observed at pH 8, where the *K*_a_ dropped from 4.5 (±1.4) × 10^5^ M^–1^ to 5.8 (±0) × 10^4^ M^–1^, corresponding to an 87.2% reduction in affinity. These data provide compelling evidence that Trp60 is essential for the coumarin-Grb2 interaction. Additionally, these data also demonstrate an increase in affinity with increasing pH, corroborating the finding that coumarin’s interaction with Grb2 is enhanced at pH values close to those found in the cytosol of cancer cells.^14^

Once our hypothesis was confirmed, we further evaluated the behavior of the protein–ligand complex through molecular dynamics. We conducted simulations in triplicates, yielding a total of 1.5 μs of structural data for each site. The trajectories from each simulation were concatenated, clustered, and analyzed using Elvim software^19^ to identify the representative conformations of each site. Briefly, this method projects the high-dimensional conformational ensemble into a 2D effective energy landscape by preserving the local structural dissimilarity between frames. In this projection (Figure 4), the regions with high state density (shown in dark red) correspond to the thermodynamic basins or “clusters” where the protein conformation is most stable and resides the longest. By extracting the centroids of these high-density regions, we identified the representative structures for each state.

**Figure 4:**
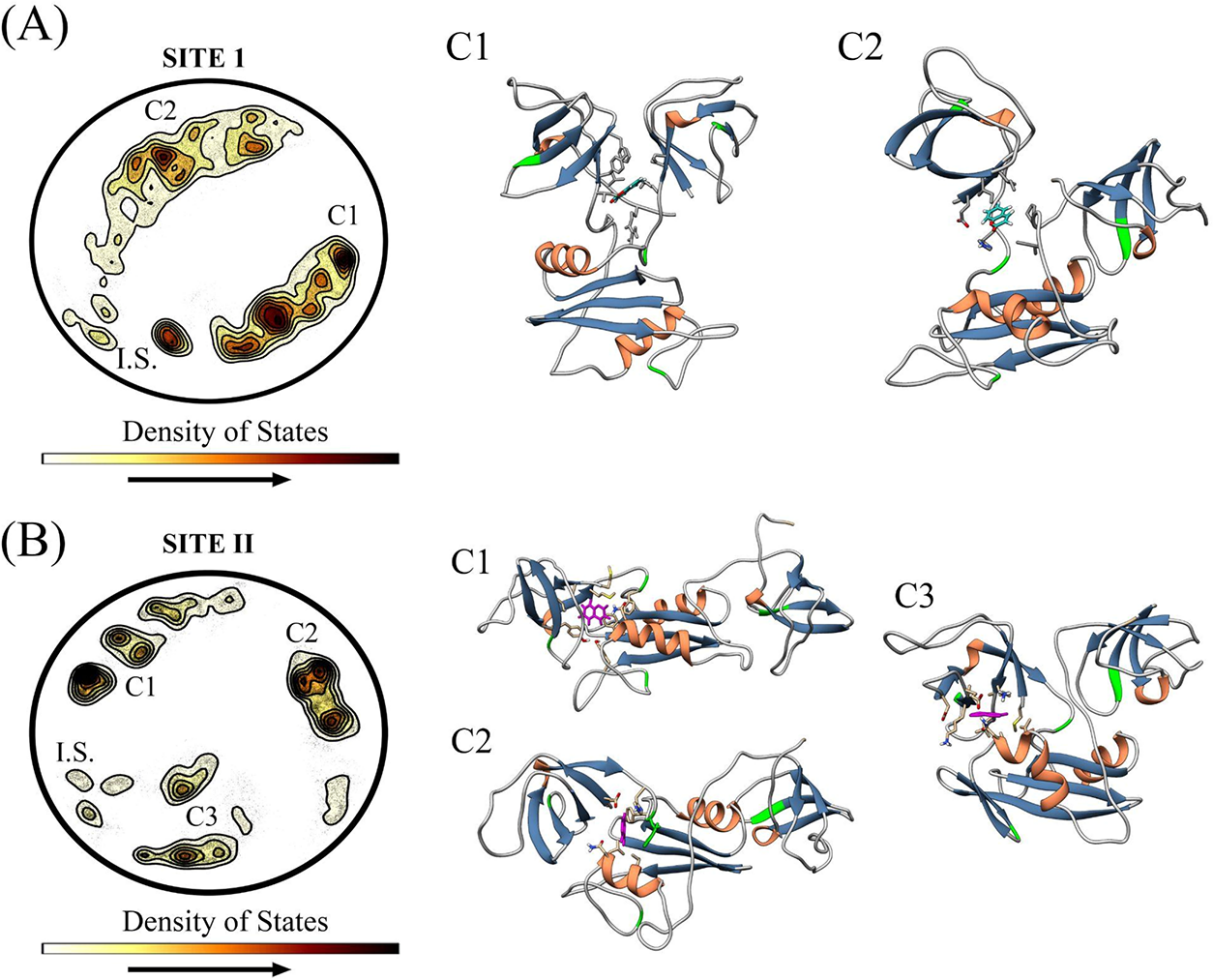
Grb2-coumarin structural clusters after molecular dynamics simulations. (A) Site I revealed two ensembles (C1 and C2), where structural rearrangements displaced the coumarin, resulting in a new site at the interface formed between the SH3 domains. (B) The dynamics showed three representative clusters with more flexible structures and interfaces formed between the SH3 and SH2 domains.

Two conformational clusters (C1 and C2) were identified for site I (Figure 4A). Cluster C1 has a Grb2 conformation highly similar to the monomer taken from PBD 1GRI,^16^ in which the SH3 domains are close to each other. This structural similarity is quantitatively supported by a RMSD of 0.991 Å between the cluster representative and the crystal structure, as illustrated in the superposition shown in Figure S7. In both cases, there are interdomain interactions between the SH3 domains, allowing coumarin to move and stabilize into the hydrophobic site formed by them. This behavior is consistent across all dynamics simulations, confirming the stabilization of coumarin in site I. As for site II, three conformational clusters were identified, with all of which showing some sort of distancing between the SH3 domains and coumarin stabilized into the SH2 pocket (Figure 4B). For both sites, the C1 clusters best align with the experimental results and are the most representative in terms of state density, exhibiting well-defined hydrophobic cavities and coumarin conformations that closely match the predictions from STD-NMR.

The average distances between coumarin and the docking site suggest a rearrangement and migration of coumarin to the SH2 pocket as the SH3 domains lose their interdomain contacts and move apart. Upon reaching site II, coumarin exhibits stabilization effects across all dynamic analyses. This observation is further supported by the average RMSD (Root Mean Square Deviation) and radius of gyration (Figures S3 and S4), which align with the structural changes observed in the projections.

Based on the observed distancing of the SH3 domains at site II through MD projections, and given the high flexibility of Grb2 in solution,^20^ we hypothesized that the increased pH promotes structural expansion and enhanced domain flexibility. This conformational shift likely facilitates the release of coumarin from site I and its subsequent binding at site II. To test this, we determined the hydrodynamic diameter (*D*_H_) of Grb2 in pH 8 using Dynamic Light Scattering (DLS) (Figure S5). For these measurements, a 1.5 mg/mL protein solution in sodium phosphate buffer was analyzed in triplicate at 20 °C. We obtained a *D*_H_ = 6.02 (±0.07) nm, representing an increase of approximately 1.2 nm compared to previously reported values at pH 7.^15^ This finding is consistent with the pH-induced expansion observed for the Grb2 dimer^13^ and likely results from the increased spatial separation of the SH3 domains. Given that these domains already exhibit dynamic “opening and closing” movements at pH 7, our results suggest that alkalization intensifies this distancing, thereby modulating the protein’s ligand-binding landscape.

Finally, bringing together all the *in vitro* and *in silico* results, a comparison of each cluster with the STD-NMR data reveals that cluster 1 from both sites is the most representative of the Grb2-coumarin complex. This conclusion was based on factors such as ensemble size, hydrophobicity, the presence of tryptophan, and epitope mapping. In site I, the binding occurs in a hydrophobic area at the interface between the SH3 domains and does not involve any direct interaction with tryptophan (Figure 5A). This finding aligns with the less accentuated fluorescence quenching data observed at pH 7. In the cluster C1 model, the protons H6 and H8 which exhibit the highest *A*_STD_ values (100% and 76.6%) are oriented toward the protein surface, maintaining contact distances <3.5Å with residues Pro59 and Phe62. In contrast, protons H3 and H7 *A*_STD_ ∼ 25% are solvent-exposed, consistent with their weak saturation transfer. Alternative clusters failed to reproduce this specific orientation. Conversely, site II is an hydrophobic pocket in the SH2 domain, where coumarin is completely buried and allows direct contact between coumarin and Trp60 (Figure 5B), which supports the more accentuated fluorescence quenching at pH 8 and higher *A*_STD_ values than at pH 7.

**Figure 5:**
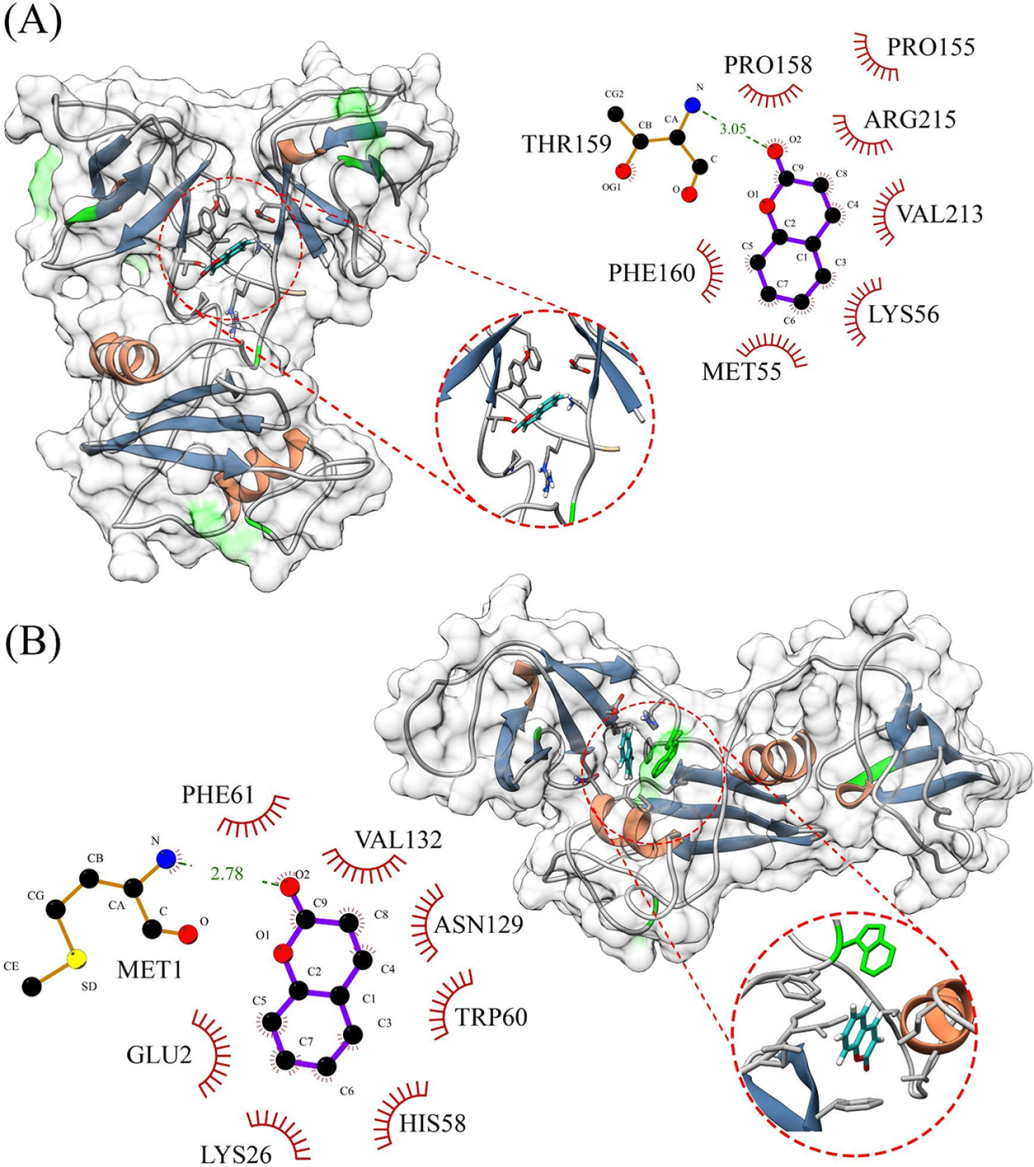
Mapping of the interaction between coumarin and Grb2 residues using the most representative cluster for each site. (A) Site I is composed of residues PHE160, MET155, LYS56, VAL213, ARG215, PRO155, and PRO158, with THR159 mediating a hydrogen bond with the oxygen atom of coumarin. There are no tryptophan residues (highlighted in green) within a distance that can be defined as a contact, which supports our hypothesis that this is the most probable binding conformation at pH 7. (B) Site II shows a predominance of hydrophobic interactions involving residues Phe61, Val132, Asn129, Trp60, His58, LYS26, and Glu2, with Met1 forming a hydrogen bond with the oxygen atom of coumarin. Unlike what was observed in (A), this site features a direct interaction with Trp60, supporting our hypothesis that this is the most likely binding conformation at pH 8, due to the pronounced fluorescence quenching observed.

## 3 Conclusion

Grb2 is an essential protein that participates in initial signaling complexes that lead to the downstream of the RAS/MAPK pathway, which is one of the main signaling pathways for cell proliferation and differentiation. In this study, we observed that the quenching pattern of tryptophan fluorescence emission differs in the interaction between Grb2 and coumarin at different pH values, suggesting that the pH increase from 7 to 8 modulates the protein’s structural conformation and, consequently, the accessibility of different binding sites. This hypothesis is supported by the DLS data, which revealed a 1.2 nm increase in the hydrodynamic diameter of Grb2 at pH 8 compared to pH 7, as well as by STD-NMR experiments, which showed enhanced coumarin epitopes at pH 8. These findings are further corroborated by an association constant (*K*_a_) 10-fold higher at pH 8. Additionally, we explored potential binding sites and found that, unlike previous findings from our group, coumarin preferentially interacts with Trp60 in the monomeric form of Grb2. This is the first study to demonstrate this interaction, as earlier work was conducted using the dimeric form of the protein, in which Trp60 is buried within the dimerization interface and thus inaccessible for ligand binding. Moreover, since the Grb2-coumarin complex exhibits higher stability at a pH close to the intracellular environment of cancer cells (pH 8), this study highlights the importance of choosing appropriate experimental conditions during in vitro testing, as choosing physicochemical conditions different from the target pathological condition may interfere with the structure and dynamics of the proteins involved and potentially affect the efficacy of a protein–ligand interaction.

## 4 Material and Methods

### 4.1 Protein Expression and Purification

Histidine-tagged Grb2 (Y160F/W60A) was purified from *Escherichia coli* BL21 (DE3) cells. In this work, we focused on the mutant Y160F because it predominantly exists as a monomer in solution. The mutation, W60A, was used to further explore the main hypothesis. A preculture of 100 mL of each protein was grown overnight and was used to inoculate 1 l of LB media supplemented with 50 μg/mL kanamycin. The cultures were allowed to grow at 37 °C and 100 rpm until the OD_600_ = 0.7. Protein expression was induced at 20 °C with 0.2 mM IPTG and incubation for 16 h with constant shaking at 100 rpm. Cells were harvested by centrifugation (3600*g* at 4 °C for 30 min), resuspended in 50 mM Tris; 100 mM NaCl; 1 mM of β-mercaptoethanol (BME) buffer at pH 8 and submitted to sonication on ice (10 cycles of 30 s comprised of 2 s pulse-ON time/1 s pulse-OFF time, 50 μm amplitude). Cell debris was removed by centrifugation (35,000*g* at 4 °C for 90 min). The soluble fraction was applied to an affinity column charged with cobalt previously loaded with the lysis buffer containing 10 mM Imidazole. Proteins were eluted by crescent Imidazole concentrations (30–500 mM) and then concentrated to 2 mL. Gel filtration was performed to change the buffer solution to 20 mM NaPi (Na_2_HPO_4_/NaH_2_PO_4_), 50 mM NaCl; 1 mM BME at pH 7 or 8 in a XK 16/70 column packed with Superdex 75 resin. Protein purity was verified by 15% SDS-PAGE.

### 4.2 Fluorescence Spectroscopy

Experiments were conducted using an ISS PC1 spectrofluorometer coupled with a Thermo Fisher Neslab RTE-221 refrigerated bath to determine the association constant (*K*_a_) and thermodynamic parameters of the interaction. Tryptophan was used as an endogenous probe, and the excitation wavelength was set to 290 nm. The emission spectra were collected over the range of 300 to 500 nm. Measurements were performed in triplicates at temperatures of 295, 299, and 303 K using 2 mL samples in a quartz cuvette with a 1 cm optical path length. The protein concentration was adjusted to 2 μM, and coumarin titrations were performed until the ratio between protein and ligand in the solution reached 1:1. The stock solution of coumarin was prepared in ethanol at a concentration of 202 μM. The total volume in the cuvette varied by approximately 3% considering the titrations.

The association constant (*K*_a_) between Grb2 and coumarin was calculated according to eq 1, assuming a two-state model for the protein–ligand interaction (FP + FL → PL), where FP represents the free protein, FL the free ligand, and PL the complex between protein and ligand.^20^

1 $$K_{a}=\frac{\left[\mathrm{PL}\right]}{\left[\mathrm{FP}\right]\left[\mathrm{FL}\right]}$$

The fraction θ of the binding sites on the protein that are occupied by the ligand is described by eq 2, where *K*_d_ is the dissociation constant, the reciprocal of the association constant $K_{d}=\frac{1}{K_{a}}$ . For a protein with *n* binding sites, the equilibrium equation is expressed as (P + *nL* ↔ PL_*n*_), and therefore, the fraction θ can be rewritten as eq 3.^21^

2 $$\theta =\frac{occupied\;binding\;sites}{total\;of\;binding\;sites}=\frac{\left[PL\right]}{\left[PL\right]+\left[P\right]}=\frac{\left[L\right]}{\left[L\right]+\left[K_{d}\right]}$$

3 $$\theta =\frac{{\left[L\right]}^{n}}{{\left[L\right]}^{n}+K_{d}}$$

By rearranging the terms and applying the logarithm to both sides, we obtain eq 4, also known as Hill equation double-log equation.^20^ The plot of $\log\left(\frac{\theta }{1-\theta }\right)$ versus log­[*L*] provides *n* as the slope, and from the intercept, we can obtain the dissociation constant *K*_d_. In this equation, since the parameter *n* value is near 1 (the unity), we assume that there is no cooperativity taken place in the interaction process so in this case the parameter *n* is considered as the number of binding sites (stoichiometry).

4 $$\log\left(\frac{\theta }{1-\theta }\right)=n\log\left[L\right]-\log K_{d}$$

The correspondence between the Hill equation and the fluorescence quenching of a protein is established through the parameter θ. The estimate of this parameter is given by the fraction of the initial fluorescence of the protein that is suppressed in the presence of the quencher (ligand). When we make this correspondence, we are indicating that the fluorescence accessible to the ligand/quencher at a specific binding site is suppressed, and the observed fluorescence comes from regions of the protein where the binding sites are unoccupied. Thus, the correspondence is structured as follows

5 $$\theta =\frac{F_{0}-F}{F_{0}}$$

Where *F*_0_ and *F* are the fluorescence intensities of the protein in the absence and presence of the quencher, respectively.

The correction for the inner filter effect was performed using eq 6, where *F*_A_ refers to the corrected fluorescence, *F*_O_ refers to the observed fluorescence, *A*_λex_ refers to the molar extinction coefficient of the ligand at the excitation wavelength, and *A*_λem_ refers to the emission wavelength used in the analysis of the data.^22^

6 $$F_{A}=F_{O}\times 10^{\left(A_{\lambda \mathrm{ex}}+A_{\lambda \mathrm{em}}\right)/2}$$

To determine the thermodynamic parameters of the interaction, fluorescence quenching experiments were conducted at three temperatures (288, 298, and 308 K). The resulting association constants (*K*_a_) were analyzed using the van’t Hoff eq (eq 7).^23^

7 $$-\ln\left(K_{a}\right)=\frac{\Delta H}{\mathrm{RT}}-\frac{\Delta S}{R}$$

The natural logarithm of *K*_a_ was plotted against the reciprocal of the absolute temperature (1/*T*) to construct a van’t Hoff plot. Standard enthalpy (Δ*H*) and entropy (Δ*S*) changes were derived from the slope $\left(\frac{-\Delta H^{{}^{\circ}}}{R}\right)$ and y-intercept $\left(\frac{\Delta S^{{}^{\circ}}}{R}\right)$ of the linear regression, respectively, where R is the universal gas constant (8.314 J · mol^–1^ · K^–1^).

Finally, the Gibbs free energy (Δ*G*) was calculated to determine if the reaction is spontaneous (eq 8).

8 ΔG=ΔH−TΔS

### 4.3 Saturation Transfer Difference by Nuclear Magnetic Resonance (STD-NMR)

The STD-NMR experiments were conducted on a Bruker Avance III 600.13 MHz spectrometer equipped with a 5 mm triple resonance cryoprobe, using a pulsed field gradient along the *Z*-axis. The spectra were collected using 600 μL samples containing 15 μM Grb2 in a buffer solution of 20 mM NaPi and 50 mM NaCl, with pH adjusted according to the purified sample, plus 10% deuterated water to determine the optimal saturation conditions. The best saturation conditions were confirmed at −1.5 ppm for on-resonance and 20 ppm for off-resonance. The saturation time was set to 2 s, with a total of 10k scans, ds = 4, and saturation power of −35 dBW. After titration with coumarin (2 mM diluted in deuterated ethanol), the spin-lock filter was adjusted to 30 ms to suppress the protein signal in the spectrum. Subsequently, the spectra were processed using Bruker TopSpin software version 4.4.1, and the epitope mapping was calculated as the ratio of the intensities of the STD spectrum to the reference spectrum according to the following equation.^18,24^

9 $$A_{\mathrm{STD}}=\frac{I_{0}-I_{\mathrm{SAT}}}{I_{0}}=\frac{I_{\mathrm{STD}}}{I_{0}}$$

where *I*_0_ is the intensity of the reference spectrum (off-resonance), *I*_SAT_ is the intensity of the saturation spectrum (on-resonance), and *I*_STD_ is the intensity of the difference spectrum (off-resonance—on-resonance). The values were normalized by multiplying the highest value by 100%, with the other values being multiplied relative to the first.

### 4.4 Dynamic Light Scattering (DLS)

DLS experiments were carried out using a Zetasizer Nano ZS90 (Malvern Panalytical) with 1 mg/mL (40 μM) at 20 °C. Data were obtained by the mean of 3 measurements comprising 10 scans each. The autocorrelation function and hydrodynamic diameter calculations were previously described elsewhere.^13^

### 4.5 Molecular Docking

The molecular docking steps of the Grb2-Coumarin complex were carried out using the three-dimensional information obtained experimentally through SAXS and molecular dynamics in a previous work.^15^ The three-dimensional structure of Coumarin was obtained from the PubChem database^25^ with the compound identification number (CID: 323). UCSF Chimera 1.7 ^26^ was used to prepare the ligand and the receptor. Molecular docking calculations were performed using UCSF Dock 6.7.^27^ The identification of the protein cavities was conducted with the SPGHEN tool, which is part of the UCSF DOCK package. During the docking procedure, the Grb2 receptor was treated as a rigid entity, while the ligand was initially configured as flexible during the GRID Score step. After this phase, both the ligand and the receptor’s interaction region were considered flexible for the AMBER recalculation. The first phase of molecular docking was evaluated using a single-grid energy scoring function (SGE), which includes van der Waals and electrostatic interactions, referred to as the Grid Score phase. Subsequently, we performed a ranking of the conformations obtained in the SGE through the Amber Recalculation Binding Energy (ASBE). During this phase, small adjustments were allowed due to the flexibility of both the receptor and the ligand. All parameter settings followed the configuration reported elsewhere.^12^

### 4.6 Molecular Dynamics Simulations

The best results obtained from molecular docking were used for the molecular dynamics portion. Molecular dynamics simulations were conducted using the GROMACS package, version 5.0.7,^28^ utilizing the GROMOS54A7 force field and the SPC water model.^29^ The topology parameters for the Coumarin molecule were obtained using the ATB server.^30^ This combination of software and parametrization is complementary, allowing for a robust calculation of the molecular interactions between the protein and the ligand. Energy minimization was performed using 50,000 steps with the steepest descent method and 5000 steps with the conjugate gradient method, both without positional restraints. System equilibration was carried out in two steps of 250 ps, initially with and then without positional restraints for the atoms of the protein and ligand. MD simulations were conducted for 500 ns in triplicate, with an integration time step of 2 fs at 298 K, a salt concentration of 0.15 M, and a pressure of 1 atm. Other parameters, such as the integrator used, barostat, thermostat, restraints, and analyses, were performed following the protocol described elsewhere.^12^

### 4.7 Energy Landscape Visualization Method (ELViM)

The Energy Landscape Visualization Method (ELViM) was used to identify and analyze the structural differences of the conformational clusters sampled from molecular dynamics (MD) trajectories. By utilizing the density of states, we located the key structural sites throughout the dynamics. The ELViM software is a multidimensional projection method developed to create intuitive representations of high-dimensional phase spaces in biomolecular contexts.^31^ By employing an internal distance metric that captures the variations among the sampled structures, the method positions each conformation as a point on a plane. This approach ensures that the partial Euclidean distances between the points accurately reflect the original dissimilarity between the conformations. A detailed description of the method, along with instructions for its implementation, can be found on GitHub (https://github.com/VLeiteGroup/ELViM).^19^ Additionally, other studies involving the GRB2 system have successfully applied this method, demonstrating its versatility and suggesting that the protein is an excellent target for conformational investigations using projection.^32^

## Supplementary Material



## Notes

Analysis of fluorescence emission spectra from the mutant monomeric Grb2 Y160F (295 and 303 K) and W60A (all temperatures) with coumarin; Average RMSD and radius of gyration plots for the three dynamics simulations of conformations C1 and C2; Grb2 Y160F DLS data in pH 8, Distance analysis between the Coumarin molecule and the Trp residues of the Grb2 protein; Structural superposition of the Site I-Cluster C1 representative conformation and the crystallographic structure of the Grb2 monomer (PDF)
