# Supplementary material for: pH-Dependent Conformational Changes in Grb2 Monomer Reveal Different Binding Sites for Coumarin: An Insight for Small Molecule Drug Discovery
Source: ACS Omega. 2026 Jan 30;11(6):9451–9. doi: 10.1021/acsomega.5c09449 (PMC12917664; doi:10.1021/acsomega.5c09449)
Supplement: Supplementary file 1 [file ao5c09449_si_001.pdf]

SUPPORTING INFORMATION

pH-Dependent conformational changes in Grb2 monomer reveal different binding sites for Coumarin: An insight for small molecule drug discovery

Giovana Casteluci[1,2]; Raphael Vinicius Rodrigues Dias[1,2]; Jéssica Andrade Tedesco[1,2]; Lucas Eduardo Gouveia[1]; Aline Sebastiane Gonçales Ramos de Oliveira[1,2]; Ícaro Putinhon Caruso[1,2]; Fernando Alves de Melo[1,2,*]

[1] Department of Physics, São Paulo State University (UNESP), Institute of Biosciences, Humanities and Exact Sciences, 15054-000, São José do Rio Preto, SP, Brazil.

[2] Multiuser Center for Biomolecular Innovation (CMIB), São Paulo State University (UNESP), Institute of Biosciences, Humanities and Exact Sciences, 15054-000, São José do Rio Preto, SP, Brazil.

Corresponding author

Fernando Alves de Melo, Department of Physics, São Paulo State University (UNESP), Institute of Biosciences, Humanities, and Exact Sciences, São José do Rio Preto, SP 15054-000, Brazil; Multiuser Center for Biomolecular Innovation (CMIB), São Paulo State University (UNESP), São José do Rio Preto, SP 15054-000, Brazil.

Email: fernando.melo@unesp.br

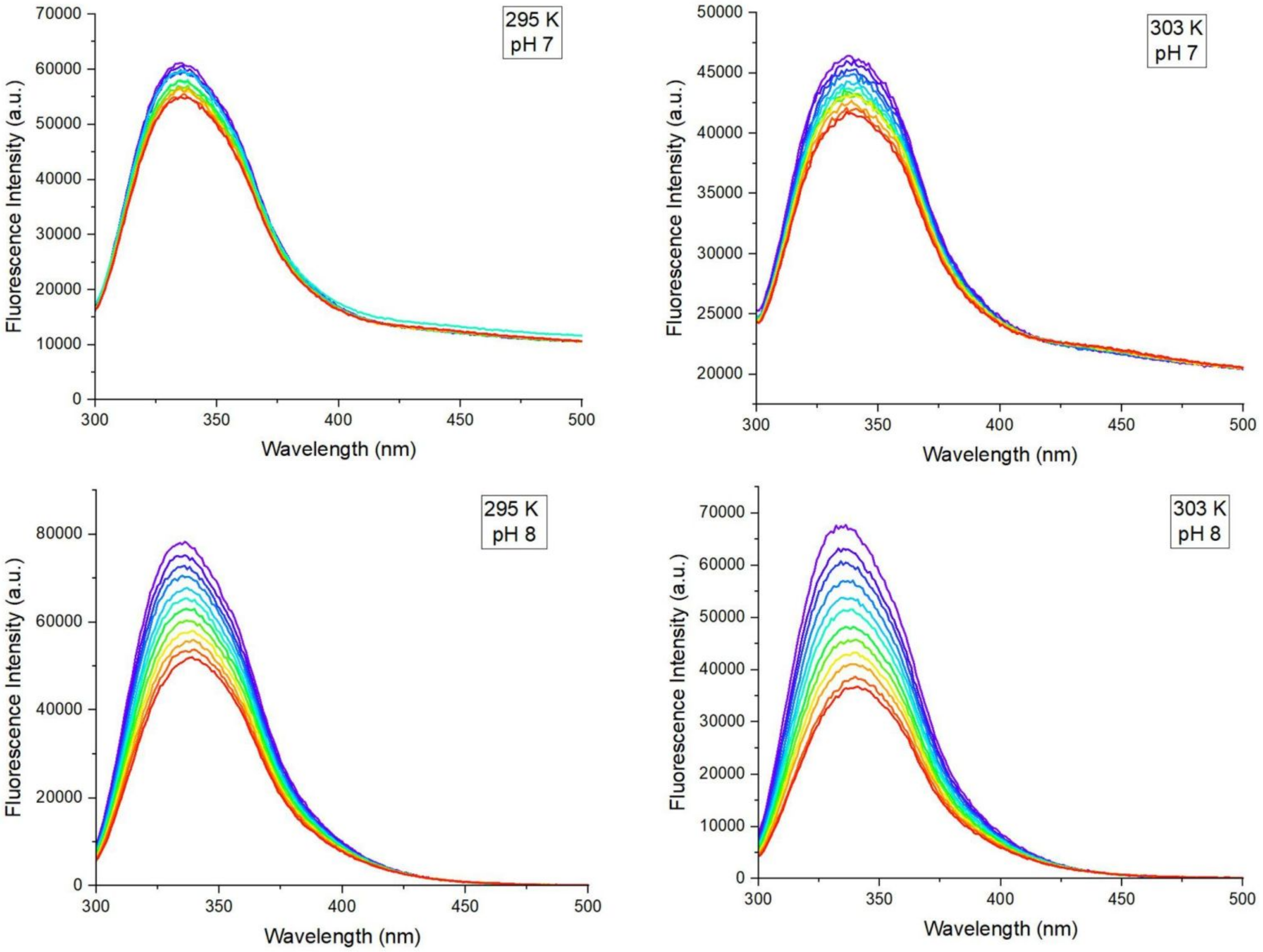

Figure S1. Analysis of fluorescence emission spectra from the monomeric Grb2 and coumarin interaction at 295 and 303 K. Coumarin was titrated until the system reached a 1:1 ratio between protein and ligand.

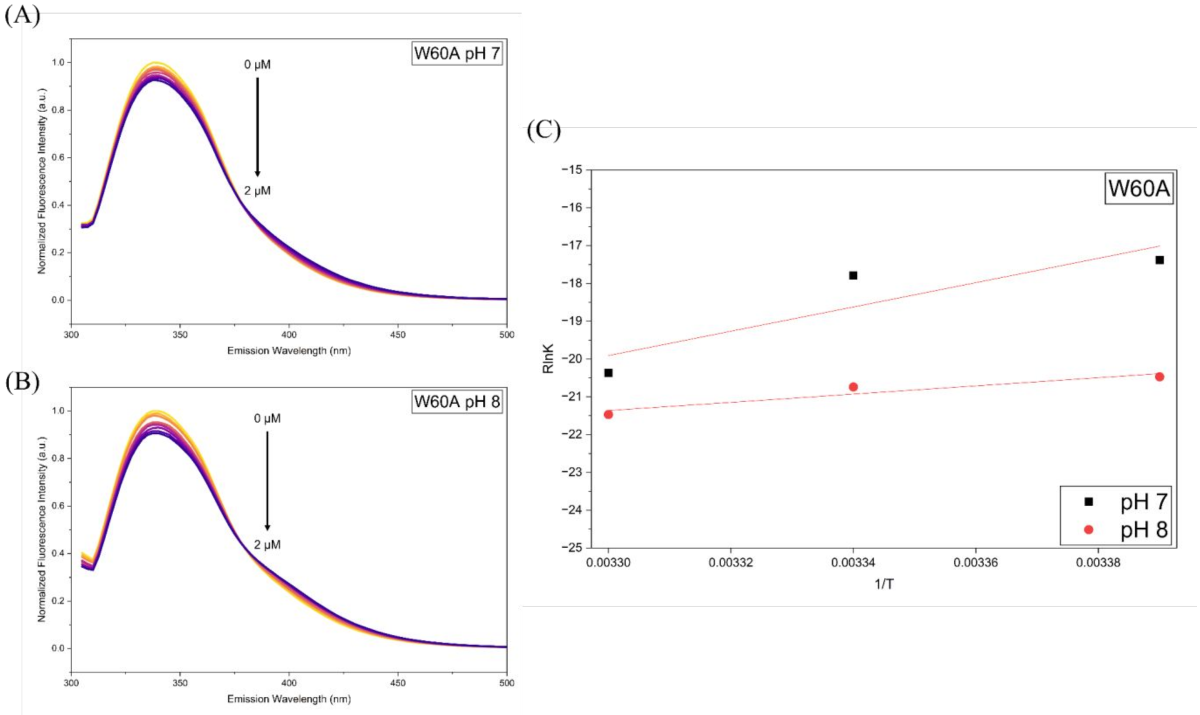

Figure S2. Analysis of fluorescence emission spectra from the monomeric Grb2 W60A and coumarin interaction. Coumarin was titrated until the system reached a 1:1 ratio between protein and ligand. Experiments were performed at 295, 299, and 303 K. Since the data showed a similar quenching pattern across all temperatures, only the results at 299 K are shown here. A-B) Fluorescence emission spectra at pH 7 and 8, respectively. C) Van't Hoff plot of both pH conditions. The plot was constructed by plotting the natural logarithm of the association constant against the reciprocal of the absolute temperature (1/T). The thermodynamic parameters were obtained from the linear regression.

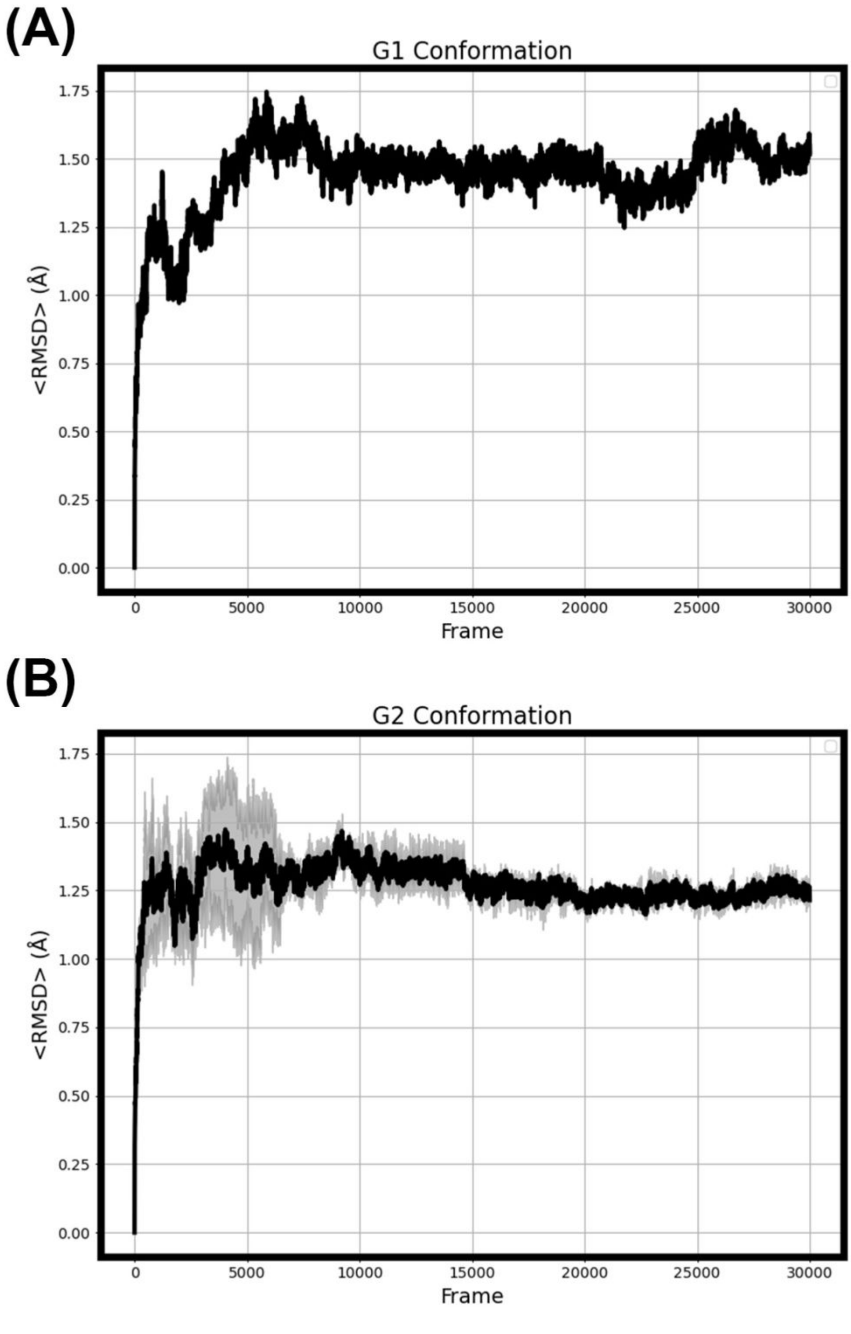

Figure S3. Average RMSD plots for the three dynamics simulations of conformations (A) C1 and (B) C2. The central line represents the mean values, while the shaded area indicates the standard deviation. In the C1 conformation, all three replicas exhibit similar behavior, indicating good stability of this conformation. In contrast, for the C2 conformation, the standard deviation is more pronounced, suggesting that conformational changes do not follow a consistent pattern. However, all replicas converge to a representative structure at the end of the simulations.

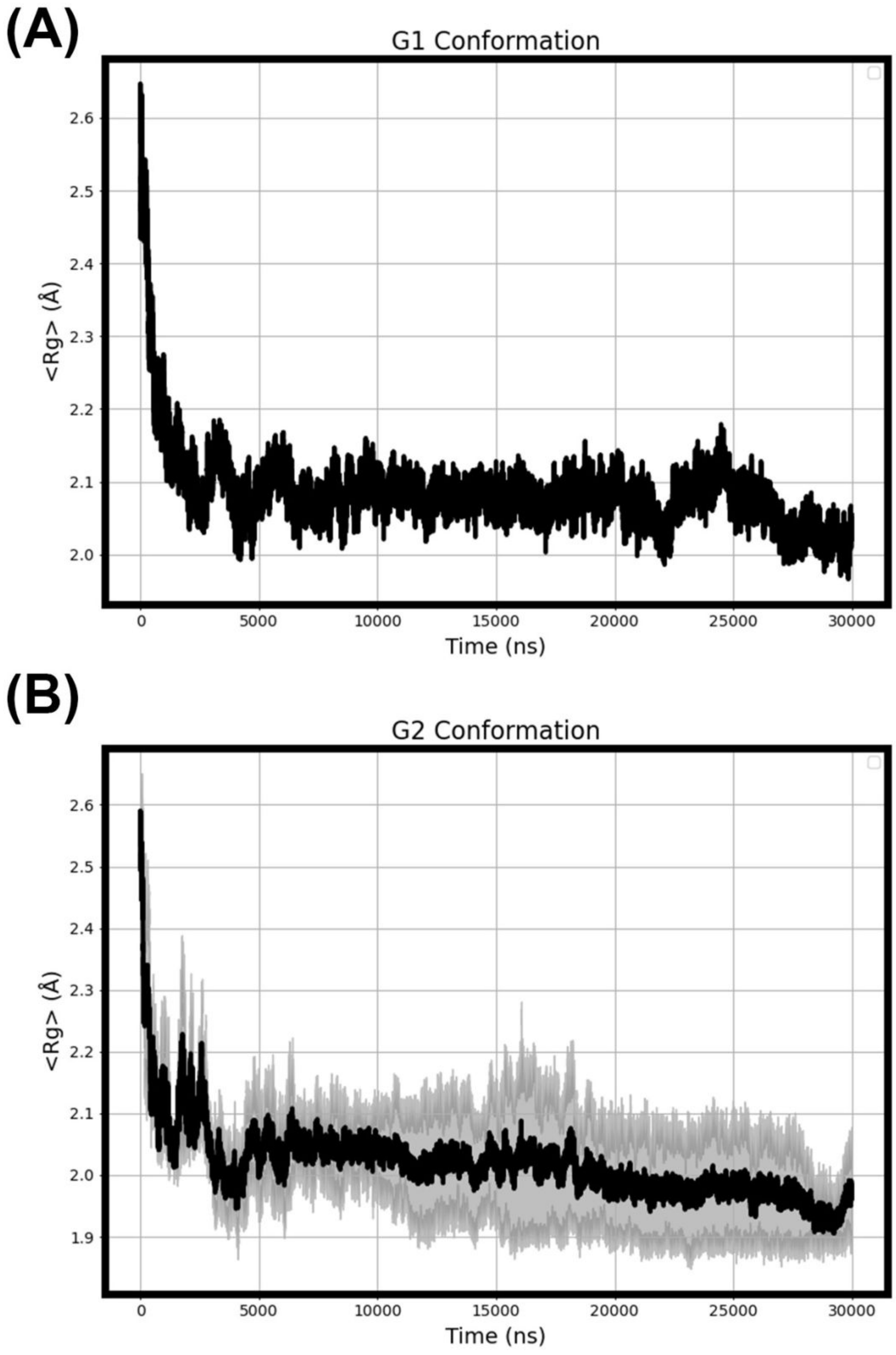

Figure S4. Average plots of the radius of gyration (Rg) for the three dynamics simulations of conformations (A) C1 and (B) C2. The central line indicates the mean values, while the shaded area corresponds to the standard deviation. Similar to the RMSD results, both conformations exhibit convergence to a stable value after roughly 50 ns. Notably, the Rg values show a compaction of both structures during the molecular dynamics, indicating that both C1 and C2 become more compact over time.

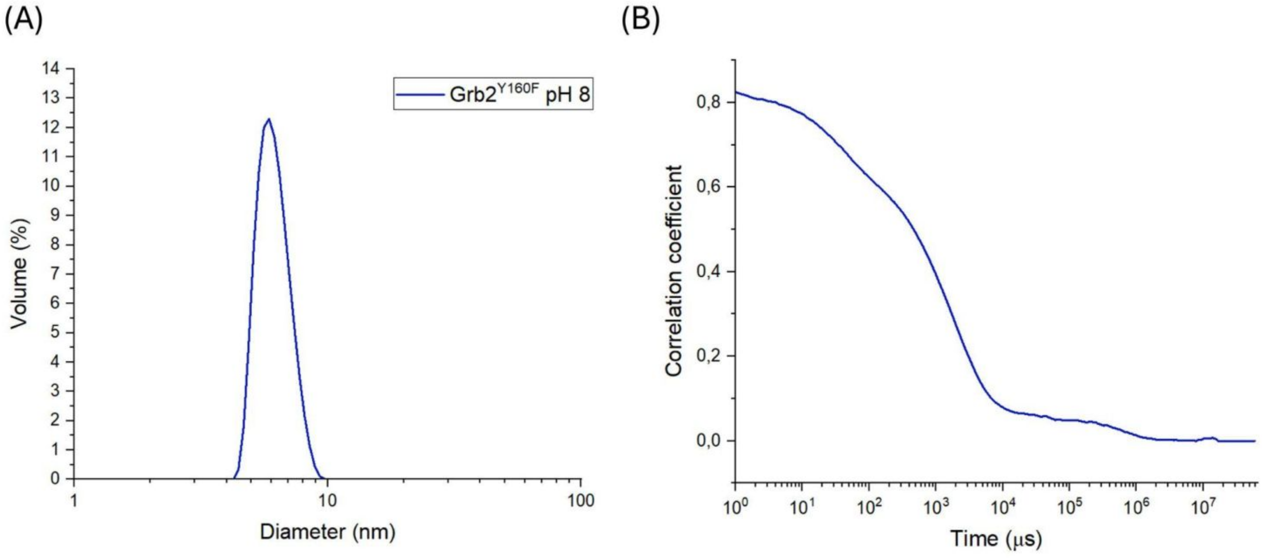

Figure S5. Particle size of Grb2^{Y160F} at pH 8 measured by DLS at 20 ºC. (A) Size distribution by volume. (B) Correlation coefficient through time.

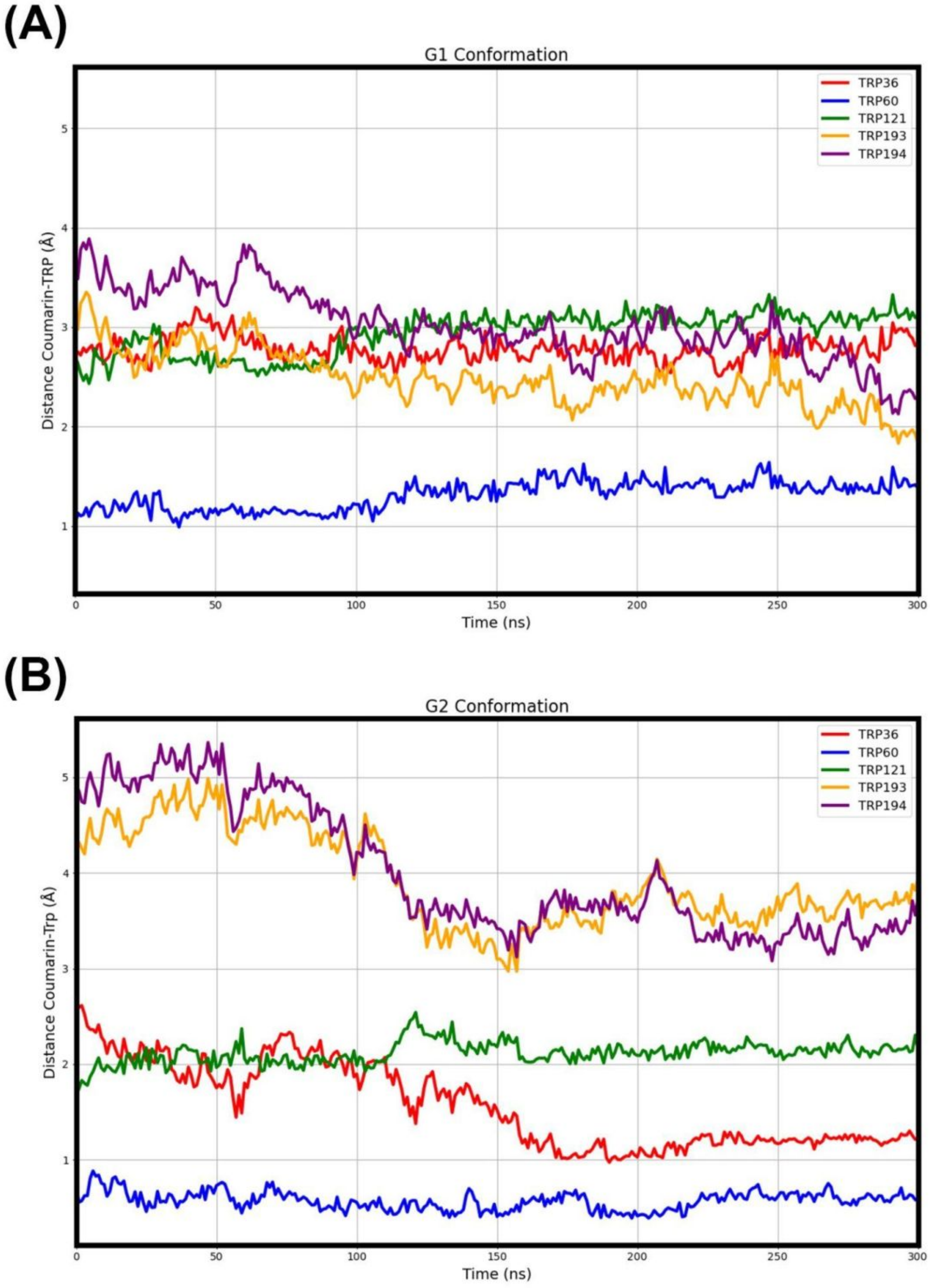

Figure S6. Distance analysis between the Coumarine molecule and the Trp residues of the Grb2 protein. In both conformations, the molecule preferentially remains near a pocket close to W60. (A) In conformation C1, the other four Trp residues are maintained at an average distance between 2 and 3 Å. (B) In contrast, for conformation C2, although W60 remains at a similar distance as in C1, the other four Trp residues are more dispersed compared to C1, indicating a greater positional variability in this conformation.

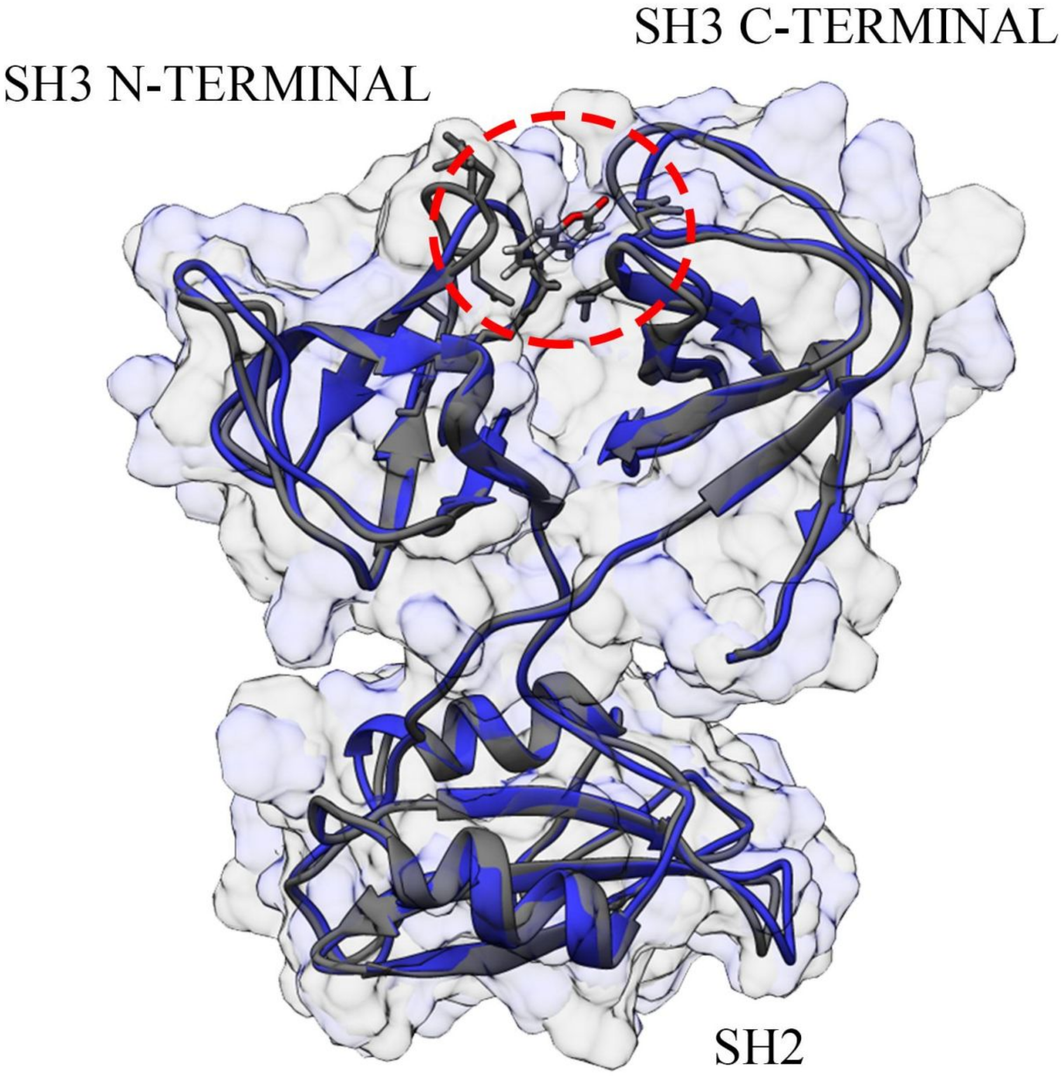

Figure S7. Structural superposition of the Site I-Cluster C1 representative conformation (gray) and the crystallographic structure of the Grb2 monomer (PDB 1GRI, blue). The dashed red circle highlights the coumarin binding site at the interface of the SH3 domains. The calculated RMSD between the two structures is 0.991 Å, demonstrating high structural conservation and confirming that the simulation accurately maintains the native fold of the protein.
